# Sociodemographic and geographic inequalities in exposure to projected hot and extreme summer days in England: A nationwide socio-spatial analysis

**DOI:** 10.1016/j.envint.2025.109351

**Published:** 2025-03

**Authors:** Jonathan R Olsen, Claire Niedzwiedz, Natalie Nicholls, Benedict W Wheeler, Frederick K Ho, Jill P. Pell

**Affiliations:** a MRC/CSO Social and Public Health Sciences Unit, School of Health and Wellbeing, University of Glasgow, UK; b School of Health and Wellbeing, University of Glasgow, UK; c European Centre for Environment and Human Health, University of Exeter Medical School, University of Exeter, UK

**Keywords:** Climate change, Inequalities, Geographical inequalities, Health, Health inequalities, Global heating

## Abstract

**Introduction:**

Climate change is a global health emergency, with extreme heat events increasing morbidity, mortality, and hospitalisations, and exacerbating health and social inequalities. Global temperatures have risen by over 1.1 °C since pre-industrial times and could reach 2.5 °C or even 4.0 °C based on current policies.

This study examined how projected climate change will vary geographically and by population sub-group in England, to assess whether these changes will affect existing health inequalities.

**Methods:**

We used small-area baseline (2001 to 2020) and climate projection data from the UK’s Met Office, linking it to area-level sociodemographic (2019) and health data (2016/17 to 2020/21), including age, socioeconomic status, ethnicity, hospital admissions, and mortality. Summated numbers of hot and extreme summer days were plotted by area-level health and sociodemographic quintiles. Models assessed the association between area-level quintiles and the projected number of hot (>30 °C) and extreme (>35 °C) summer days under 2.5 °C and 4.0 °C global heating scenarios.

**Results:**

There were clear geographical variations in experiencing hot and extreme summer days, with the South of England seeing the highest increases. The pattern of projected temperature increases does not align with typical health inequalities, as less deprived areas will experience more hot and extreme summer days, while areas with larger ethnic minority populations will face a greater number.

**Conclusion:**

Climate adaptation strategies are needed to two levels: (1) locally to support vulnerable population groups in coping with global heating; and (2) national and global strategies to prevent global temperatures from exceeding 2.5 °C or reaching 4.0 °C, given the severe population-level health risks such increases would entail.

## Highlights

• Climate change has been declared a global health emergency.
• Investigated geographical and sociodemographic effect of climate change projections.
• Geographical patterning of projected hot and extreme summer days.
• South England and areas characterised by low deprivation have greater impact.
• Areas with a greater minority ethic population will experience a greater impact.

## Graphical abstract

**Figure:**
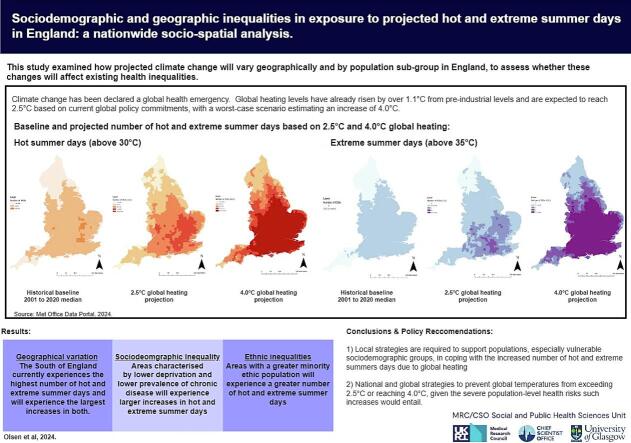

## 1 Introduction

The rise in global temperatures and the increased frequency of extreme climate events, driven by greenhouse gas emissions, pose an alarming global threat (Lee et al., 2023). Climate change has been declared a global health emergency due to its significant risks to human health and wellbeing, including from increased frequency and severity of heatwaves (McCoy et al., 2014). There is substantial evidence demonstrating the health impacts of extreme heat events and heatwaves, including increased morbidity, mortality, and healthcare needs, as evidenced by hospitalisations (Carleton and Hsiang, 2016, Li et al., 2015).

Regarding mortality, cardiovascular disease has been identified as the leading cause of death during heatwaves, followed by respiratory diseases, including COPD, and cerebrovascular disease as the third most common cause (Ebi et al., 2021). Hospital admissions for various primary causes, including respiratory and cardiovascular diseases, ischemic stroke, and cerebrovascular effects, have been shown to rise in the short-term following a heatwave (Carleton and Hsiang, 2016, Green et al., 2010, Lin et al., 2009).

The temperature threshold for the health impacts of extreme heat events can vary by location. For example, the threshold for increased respiratory admissions is 28.9 °C in New York but 23 °C in London (Ye et al., 2012). In the USA, hospital admissions for respiratory and cardiovascular diseases increase with each degree Celsius above 29 °C. Specifically, respiratory conditions see a 2.7–3.1 % same-day increase in hospital admissions per degree Celsius above 29, while cardiovascular conditions experience a 1.4–3.6 % increase in lagged hospital admissions (Lin et al., 2009). These differences in the effects of climate on health between areas, and based on the area-specific temperature increase, highlight the importance of integrating geographical perspectives on heat-related climate analyses.

The projected rise in global temperatures from pre-industrial levels is expected to exponentially worsen health outcomes worldwide (Deschenes, 2014, Gasparrini et al., 2017). However, the impact of heat and subsequent health outcomes will vary within and between countries and continents. To mitigate rising temperatures, the 26th United Nations Climate Change Conference in Glasgow (COP26), attended by 120 world leaders, reaffirmed the Paris Agreement's goal of limiting global heating to 2.0 °C above pre-industrial levels (United Nations, 2023). Despite these efforts, global temperatures continue to rise, with all days in 2023 exceeding 1.1 °C above pre-industrial levels (UK Health Security Agency, 2023). Research suggests that the Paris Agreement's target will not be met, and global heating could reach 4.0 °C above pre-industrial levels by 2060 (Betts et al., 2021).

Rising global temperatures and extreme heat events have the potential to exacerbate existing health and social inequalities, as certain population groups may be more susceptible to negative health consequences. Elderly individuals, children, those of lower socioeconomic status, outdoor workers, and minority ethnic groups have been identified as particularly vulnerable during heatwaves (Li et al., 2015). For older individuals, increased multi-morbidity, diminished sweating ability, and a higher likelihood of living alone, without access to help, increase their vulnerability. Children younger than four years are at greater risk due to their lower heat loss compared to adults and inability to regulate their body temperature behaviourally (Ebi et al., 2021).

The relationship between individual racial and ethnic identity and heat-associated morbidity and mortality is complex. While some evidence suggests that Black individuals are more vulnerable and Asians less so than other ethnicities, the reasons for these differences are not fully understood (Gronlund, 2014). Factors such as lower income, poorer health, substandard housing (including lack of air conditioning), and outdoor working conditions contribute to these disparities (Gronlund, 2014). The geographical distribution of urban summer heat island effects in the USA shows that areas with higher proportions of people of colour and lower-income populations experience worse conditions, both nationally and within major urban areas (Hsu et al., 2021). Heat vulnerability by sociodemographic characteristics, including ethnicity, in England has not been assessed.

This study aims to describe the likely socio-spatial impact of extreme heat events in England, UK, based on global heating projections for small geographical areas, considering current health burdens and sociodemographic characteristics.

## 2 Methods

### 2.1 Setting and spatial extent

This study used baseline climate data and future climate projections across England, UK. England has a total population of 57 million (mid-2022 population estimates) (Office for National Statistics, 2024) and covers an area of 130,279 km^2^. Population density is higher in the south of the country with 74 % (42 million) of the population located in the South-East, South-West and London regions. The mean 2022 air temperatures for England by season were: winter 5.7°c (min: 2.6°c; max: 8.8°c), spring 9.7°c (min: 5.2°c; max: 14.3°c), summer 11.9°c (min: 11.7°c; max: 22.5°c), and autumn 10.9°c (min: 8.4°c; max: 15.6°c) (Met Office, 2024).

Data were analysed by Middle-layer Super Output Area (MSOA). MSOAs are small area census units (n = 6,856) that comprise between 2,000 and 6,000 households and have a usually resident population between 5,000 and 15,000 (mean c.7,800) persons (Office for National Statistics, 2022).

### 2.2 Climate change projections

Data on two heat exposures with implications for public health were extracted from the online portal of the Met Office (Met Office Climate Data Portal, 2024); the UK’s meteorological service:

(i) Number of hot summer days: annual number of days where the maximum daily temperature is above 30 °C.
(ii) Number of extreme summer days: annual number of days where the maximum daily temperature is above 35 °C.

The thresholds of 30 °C for Hot Summer Days and 35 °C for Extreme Summer Days, defined by the UK Met Office, were selected due to their significant health, social, and economic impacts, including infrastructure disruption, increased morbidity, and risks to public health (Hanlon et al., 2021, LCCP, 2021, Ramsey et al., 2024). In the UK, the Heat-Health Alert system developed by the UK Health Security Agency (UKHSA) and the Met Office sets temperature thresholds for medium impact levels, defined as conditions where mortality rates across the UK have already exceeded 20 %. These thresholds are set at 30 °C for most of the UK and 32 °C for London (UK Health Security Agency, 2024). Although there is a 2 °C difference between London and the rest of the UK, and only England is included in our analysis, it remains essential to establish and use clear, policy-relevant, and measurable indicators (Murage et al., 2024a). Their use in this study ensures alignment with national projections, facilitates policy translation, and strengthens the public health applicability of the findings.

The Met Office data included baseline values as well as projected values for two future global heating scenarios suggested in the UK Health Security Agency’s Health Effect of Climate Change in the UK: 2023 report (UK Health Security Agency, 2023):

(i) Baseline – median annual data for 2001 to 2020; a period 0.87 °C warmer than pre-industrial temperatures.
(ii) 2.5 °C increase from pre-industrial temperatures – mid-range estimate of the likely impact of current global policy commitments.
(iii) 4 °C increase from pre-industrial temperatures – high-end estimate of the likely impact of current global policy commitments.

Annual hot summer days were categorised as: 1 or less, 2 to 5, 6 to 10, 11 to 15, and 16 or more. Annual extreme summer days were categorised as: 0, 0.1 to 0.5, 1, 2, and 3 or more.

### 2.3 Area-level sociodemographic data

Socioeconomic status: Deprivation quintile (most to least deprived) was assigned to MSOAs using the Index of Multiple Deprivation (IMD) Score 2019 (UK Government, 2019) (Supplementary Fig. 1 provides data mapped across England). The IMD comprises the following seven domains of deprivation which are combined and weighted: Income (weighting) (22.5 %), employment (22.5 %), health deprivation and disability (13.5 %), education, skills and training (11.5 %), crime (9.3 %), barriers to housing and services (9.3 %), and living environment (9.3 %).

**Fig. 1:**
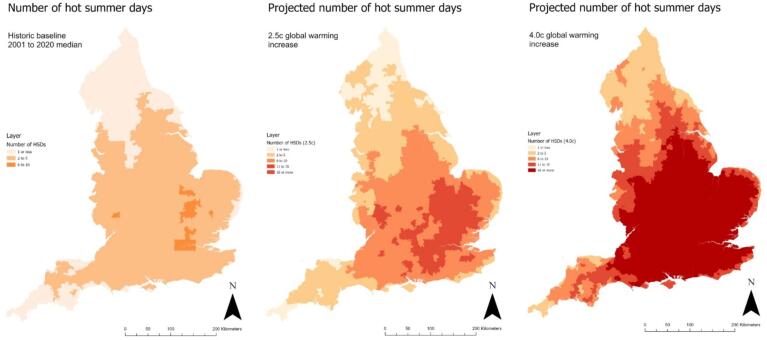
Baseline and projected number of hot summer days (temperature exceeds 30 °C) for 2.5 °C and 4.0 °C global heating increase by MSOA, England.

**Fig. 2:**
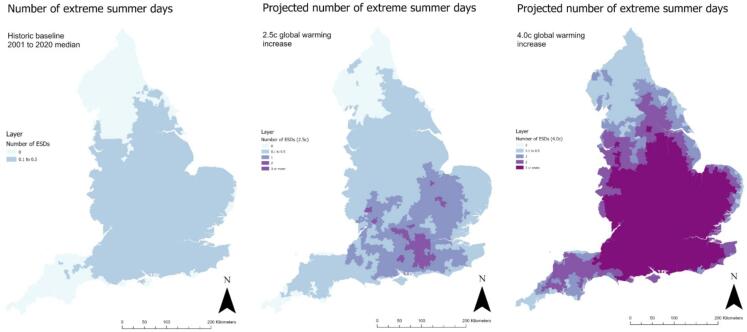
Baseline and projected number of extreme summer days (temperature exceeds 35 °C) for 2.5 °C and 4.0 °C global heating increase by MSOA, England.

Minority ethnic population: The proportion of people stating an ethnicity other than “white” in the 2011 Census were summarised for each MSOA. The ethnicity category options in the 2011 census were: White, Mixed and multiple ethnic group, Asian or Asian British, Black or African or Caribbean or Black British, and other ethnic group (Office for National Statistics (ONS), Census 2011).

Limiting long term illness or disability (LLTI): The percentage of people who responded “yes: a little or a lot” to the question “Are your day-to-day activities limited because of a health problem or disability which has lasted, or is expected to last, at least 12 months?” in the 2011 Census were summarised for each MSOA.

Age profile: Mid-year 2022 MSOA level data by single year of age were obtained and data grouped into the following categories: under 5 years, 6 to 15 years, 16 to 64 years (working age), and 65 plus years (older people) (Office for National Statistics, 2024).

### 2.4 Area-level health indicators

A number of area-level health indicators were obtained from the UK Government’s Office for Health Improvement and Disparities that provide a number of local health and public health data for MSOAs (Office for Health Improvement & Disparities, 2024). The area-level health indicators provide standardised emergency hospital admission rates for chronic obstructive pulmonary disease (COPD) and coronary heart disease (CHD) and standardised mortality ratios for causes considered preventable (under 75 years), circulatory disease (under 75 years and all age), CHD, respiratory diseases, and stroke (Table 1). Area-level quintiles were derived for each health-indicator to classify MSOAs by the most and least prevalent.

**Table 1:** Area-level health indicators ()).

**Variable**	**Description**	**Age and year**
**Standardised hospital admissions**		
Chronic obstructive pulmonary disease (COPD) hospital admissions.	Emergency hospital admissions for chronic obstructive pulmonary disease (COPD), standardised admission ratio	All persons, All Ages, 2016/17-20/21.
Coronary heart disease (CHD) hospital admissions.	Emergency hospital admissions for coronary heart disease, standardised admission ratio	All persons, All Ages, 2016/17-20/21.

**Standardised mortality ratios**		
Deaths from causes considered preventable (under 75 years).	Deaths from causes considered preventable, under 75 years, standardised mortality ratio	< 75 yrs, 2016 to 2020.
Deaths from circulatory disease (all ages).	Deaths from circulatory disease, all ages, standardised mortality ratio	All persons, All Ages, 2016 to 2020.
Deaths from coronary heart disease (CHD) (all ages).	Deaths from coronary heart disease, all ages, standardised mortality ratio	All persons, All Ages, 2016 to 2020.
Deaths from respiratory diseases, (all ages).	Deaths from respiratory diseases, all ages, standardised mortality ratio	All persons, All Ages, 2016 to 2020.
Deaths from stroke (all ages).	Deaths from stroke, all ages, standardised mortality ratio	All persons, All Ages, 2016 to 2020.

## 3 Spatial analysis

### 3.1 Computing small area climate projections

The baseline and projected climate data, which were provided on a 12 km grid covering England, were plotted within GIS software ArcGIS Pro v2.9.5. Hot and extreme summer days data were then summarised from these gridded values to MSOAs (mean values) using the ‘*summarise within*’ analysis tool (average MSOA area: 19 km). Following this spatial analysis, each MSOA was assigned the derived numbers of hot and extreme summer days at baseline and for the 2.5 °C and 4.0 °C global heating scenarios. All climate data was obtained from the UK Met Office, including the annual number of hot and summer days at baseline and for the two global heating scenarios: 2.5 °C and 4 °C. No climate modelling was conducted by the authors.

### 3.2 Analysis

Analyses assume that current/baseline socio-spatial patterns and population numbers remain constant into the future.

i. Climate projections by geographical areas and region.

The number of annual hot and extreme summer days were linked to each MSOA level and plotted across England for the baseline situation and the 2.5 °C and 4.0 °C global heating increase scenarios. Baseline and projected annual number of hot and extreme summer days, as well as change from baseline, were calculated for English administrative region (North East, North West, Yorkshire and the Humber, South West, West Midlands, East Midlands, South East, East of England, and London) to provide a description of the geographical distribution and patterning of current climate and climate change.

ii. Climate projections by population demographics.

Small area population age data were summarised for each MSOA, as the numbers aged 5 years and under, 6 to 15 years, 16 to 64 years and 65 years and over. The baseline and projected climate data at MSOA level were applied to these age categorises then summated over the whole of England to provide the current and predicted number of hot and extreme summer days, and change in these, by age-group. Chi Squared tests were performed to explore the differences by age-group.

iii. Inequalities in climate projections by socioeconomic status and health indicators.

Health and socioeconomic quintiles, and current and projected climate data were linked at the MSOA level then summated across the whole of England. Health and socioeconomic quintiles, derived from area-level indicators (Table 1), are used as an independent variable to assess whether heat exposure differs across areas of varying health and social need under baseline and global warming scenarios. The mean number of hot and extreme summer days (annual number) of a 2.5c and 4.0c global heating temperature increase are described by area-level emergency hospital admissions, mortality, and sociodemographic characteristics. The baseline and projected climate data are summated by socioeconomic status and health indicator quintiles to explore changes in the current and predicted number of hot and extreme summer days, as well as presented across quintiles for 2.5c and 4.0c global heating temperature increases.

To investigate potential associations between the socioeconomic and health indicators of an area and the median number of (extreme) hot days that area might experience under various temperature increase scenarios, Bayes-York-Molliere (Besag et al., 1991) models were used (to account for spatial autocorrelation) calling Poisson: the outcome being a rounded count of the median predicted number of (extreme) hot days. The results of these models indicate if areas with certain socioeconomic and/or health need would (continue to) be more likely to experience hot or extreme summer days, compared to their counterparts.

Models were performed individually for each variable. Analyses was performed using StataMP 18 and R version 4.4.0 (R Core Team, 2024) using the *CARBayes*(Lee, 2013) package.

## 4 Results

### 4.1 Overall impact

Between 2001–2020 (baseline), there was an average of 2.9 hot summer days (over 30 °C) per annum in England. If the mean temperature were to increase by 2.5 °C or 4.0 °C, this would increase 1.5- or 5-fold to 7.1 or 17.3 days respectively (Table 2). The number of extreme (over 35 °C) summer days would also increase from 0.2 days to 0.4 or 4.8 days respectively: the latter representing a 13-fold increase on current numbers.

**Table 2:** Projected annual number of hot and extreme summer days based on 2.5 °C and 4.0 °C global temperature increases by English region and change from baseline.

**Region**	Hot Summer Days (HSD)*				**Extreme Summer Days (ESD)^**			
	**Baseline (2000 to 2021)**	**2.5 °C increase (% increase from baseline)**	**4.0 °C increase (% increase from baseline)**	**Increase in HSD from 2.5 °C to 4.0 °C (%)**	**Baseline (2000 to 2021)**	**2.5 °C increase (% increase from baseline)**	**4.0 °C increase (% increase from baseline)**	**Increase in ESD from 2.5 °C to 4.0 °C (%)**
**North East**	0.5	1.1 (120)	4.2 (740)	3.1 (282)	0.0	0 (0)	0.4 (0)	0.4 (0)
**North West**	1.4	2.9 (107)	8.2 (486)	5.3 (183)	0.0	0.1 (0)	1.1 (0)	1 (1000)
**Yorkshire and The Humber**	1.6	3.9 (144)	11.4 (613)	7.5 (192)	0.1	0.1 (0)	1.4 (1300)	1.3 (1300)
**South West**	2.1	5.5 (162)	15.2 (624)	9.7 (176)	0.1	0.4 (300)	2.5 (2400)	2.1 (525)
**West Midlands**	3.0	6.7 (123)	17.5 (483)	10.8 (161)	0.2	0.4 (100)	3.3 (1550)	2.9 (725)
**East Midlands**	3.1	7.5 (142)	17.9 (477)	10.4 (139)	0.2	0.4 (100)	3.3 (1550)	2.9 (725)
**South East**	3.3	8.9 (170)	21.4 (548)	12.5 (140)	0.2	0.7 (250)	3.6 (1700)	2.9 (414)
**East of England**	3.8	9.6 (153)	22.1 (482)	12.5 (130)	0.2	0.5 (150)	3.6 (1700)	3.1 (620)
**London**	5.5	12.6 (129)	27.7 (404)	15.1 (120)	0.3	0.7 (133)	4.4 (1367)	3.7 (529)
**England**	**2.9**	**7.1 (145)**	**17.3 (497)**	**10.2 (144)**	**0.2**	**0.4 (100)**	**2.8 (1300)**	**2.4 (600)**

*annual number of days above 30 °C;^annual number of days above 35 °C.

Between 2001–2020 (baseline), no-one in England experienced more than 10 hot, or more than one extreme, summer days annually. With a 2.5 °C increase in mean temperature, 16 million people (29.5 % of the population) would experience more than 10 hot summer days annually and 3.2 million (6.0 %) would experience more than one extreme summer days (Table 3). With a 4.0 °C increase, 42.7 million (78.8 %) would experience more than 10 hot days and 45.3 million (83.5 %) would experience more than one extreme summer day (Table 3).

**Table 3:** English population (in thousands) experiencing hot and extreme summer days (annual number) in millions at baseline and for a 2.5 °C and 4.0 °C global heating temperature increase, by age group.

**Annual num of days**	**Under 5**			**5 to 15**			**16 to 64**			**65 and over**			**Total**		
	**Baseline**	**2.5 °C**	**4.0 °C**	**Baseline**	**2.5 °C**	**4.0 °C**	**Baseline**	**2.5 °C**	**4.0 °C**	**Baseline**	**2.5 °C**	**4.0 °C**	**Baseline**	**2.5 °C**	**4.0 °C**
**(a) Hot summer days (days > 30 °C) (0,000 s)**															
1 or less	349	93	1	903	238	2	4,268	1,192	9	1,588	453	4	7,108	1,975	16
2 to 5	2,069	824	134	5,155	2,081	345	23,963	9,831	1,701	7,628	3,363	643	38,815	16,099	2,823
6 to 10	488	1,077	452	1,091	2,683	1,139	5,692	12,414	5,277	1,045	4,014	1,775	8,316	20,187	8,644
11 to 15	0	913	398	0	2,146	1,001	0	10,487	4,804	0	2,431	1,658	0	15,977	7,861
16 plus	0	0	1,921	0	0	4,661	0	0	22,132	0	0	6,180	0	0	34,895

**(b) Extreme summer days (days > 35 °C) (0,000 s)**															
0	423	104	1	1,093	268	2	5,238	1,356	9	1,917	539	4	8,672	2,267	16
0.5 or less	2,472	1,673	174	6,029	4,178	450	28,570	19,446	2,196	8,310	6,339	830	45,381	31,636	3,651
1	11	950	265	26	2,252	681	115	11,061	3,179	34	2,829	1,162	186	17,092	5,287
2	0	180	526	0	452	1,305	0	2,060	6,172	0	554	2,009	0	3,245	10,013
3 or more	0	0	1,940	0	0	4,710	0	0	22,366	0	0	6,255	0	0	35,272
**(c) Total population (0,000 s)**															
Total	2,906			7,149			33,923			10,260			54,239		

### 4.2 Geographical regions

Currently, there is a spatial gradient across England, whereby the number of hot and extreme summer days per annum is highest in the South-East and lowest in the North-West (Fig. 1&2, Table 2). The absolute increase in both would follow the same pattern, with a higher number of additional hot and extreme summer days in the South-East. As a result, London residents would experience an increase from 5.5 hot and 0.3 extreme sunny days currently to 12.6 and 0.7 days respectively following a 2.5 °C increase, and 27.7 and 4.4 days respectively following a 4.0 °C increase (Table 2).

### 4.3 Age group

Under a 2.5 °C global heating scenario, 1 million (31.4 %) children under 5 years of age would experience more than 10 hot days per annum (Table 3), as would 12.6 million (30.8 %) of those aged 5–64 years (age group aggregate) and a slightly lower proportion of those 65 years of age or older (2.4 million (23.7 %), p < 0.001) (Supplementary Table 1). The numbers experiencing more than one extreme summer day would be 0.2 million (6.2 %), 2.5 million (6.1 %) and 0.6 million (5.4 %) respectively (p < 0.001).

Under a 4.0 °C scenario, 2.3 million (79.8 %) children under 5 years would experience more than 10 hot days, compared with 32.6 million (79.4 %) of those aged 15–64 years, and 7.8 million (76.5 %) of those 65 years of older (p < 0.001). The numbers experiencing more than one extreme summer day would be 2.5 million (84.9 %), 34.6 million (84.1 %) and 8.3 million (80.5 %) respectively (p < 0.001) (Supplementary Table 1).

### 4.4 Pre-existing long-term health conditions

**Fig. 3:**
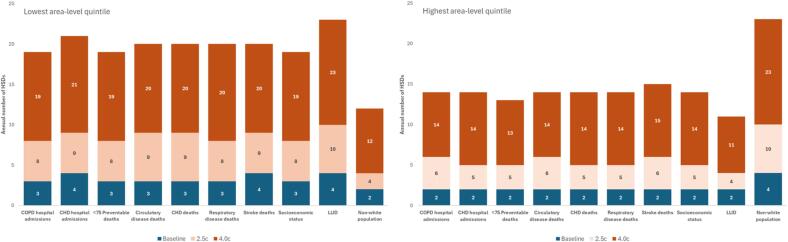
Baseline and projected number of hot summer days by area-level rates of hospital admissions, mortality ratios and sociodemographic factors for a 2.5c and 4.0c global heating increase: lowest & highest condition specific quintiles reported (Extreme summer days shown in Supplementary Fig. 2).

**Fig. 4:**
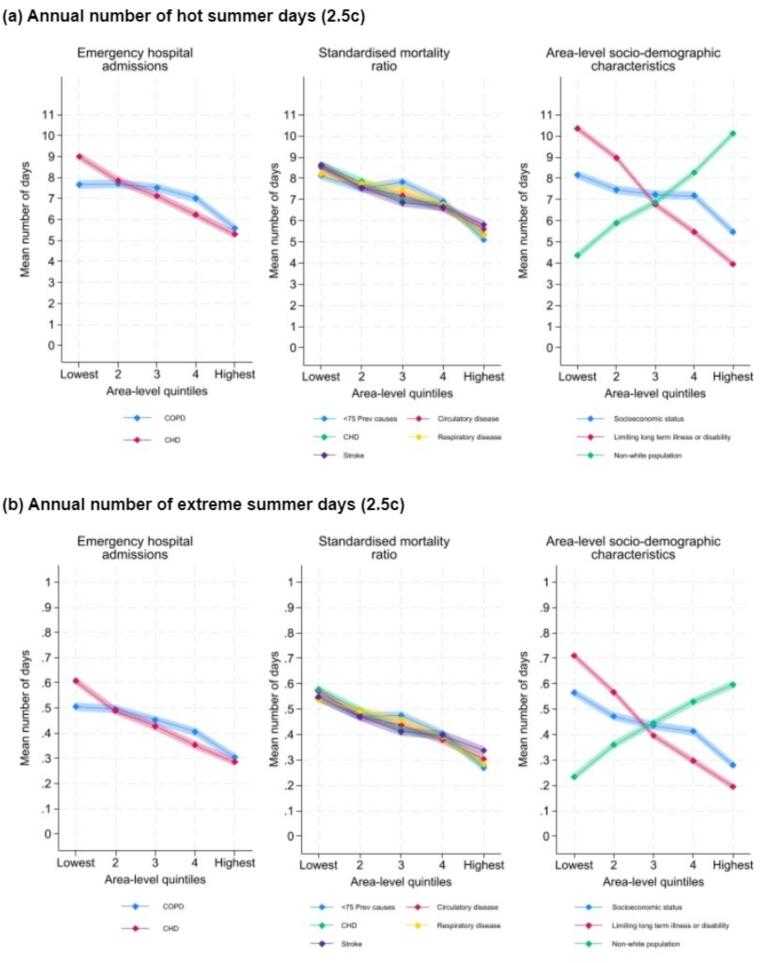
Mean number of hot and extreme summer days (annual number) of a 2.5c global heating temperature increase by area-level emergency hospital admissions, mortality, and sociodemographic characteristics. (Note: hot and extreme summer days for a 4.0 °C global heating increase are within Supplementary Fig. 3).

In the 2.5 °C global heating scenario, the additional number of hot summer days would be 33 % higher in areas in the lowest quintile for COPD hospitalisations compared with areas in the highest quintile; and 80 % higher in areas in the lowest quintile for coronary heart disease hospitalisations (Fig. 3, Supplementary Table 2). In the 4 °C global heating scenario, the figures would be 35 % and 58 % respectively. The same direction of effect was observed for all of the mortality metrics. For example, following a 2.5 °C temperature increase, there would be nine additional hot summer days in areas in the lowest quintile for coronary heart disease mortality compared with five in areas in the highest quintile (Fig. 4). Data for the number of extreme summer days are shown in Supplementary Table 3.

### 4.5 Socioeconomic status and limiting long term illness or disability (LLID)

Areas in the lowest quintile for socioeconomic status (least deprived) would experience 5 additional hot summer days in the 2.5 °C global heating scenario and 16 in the 4 °C scenario. This compared with 3 and 12 days, respectively in areas in the most deprived quintile (Fig. 3, Supplementary Table 2). Similarly, areas in the lowest quintile of LLIDs would experience 6 additional hot summer days in the 2.5 °C global heating scenario and 19 in the 4 °C scenario, which is slightly higher than areas with the lowest socioeconomic status.

### 4.6 Ethnicity

Areas with the highest proportion of minority ethnic residents would experience a higher number of hot summer days than areas with the lowest proportion in both the 2.5 °C (10 versus 4 days) and 4 °C (33 versus 16 days) global heating scenarios (Fig. 3).

### 4.7 Associations between health and sociodemographic area indicator quintiles and projected numbers of hot and extreme summer days

Fig. 4 presents the mean annual number of hot and extreme summer days by area-level quintiles for emergency hospital admissions, mortality ratios and area-level socio-demographic characteristics for a 2.5 °C global heating scenario (Supplementary Fig. 3 shows data for a 4.0 °C global heating scenario). Table 4 presents the model outputs assessing the likelihood of differences in hot and extreme summer days between area-level quintiles for each health and sociodemographic variable. The data show that areas with higher populations of ethnic minority groups are more likely to experience hot and extreme summer days (Table 4). Areas with greater populations reporting a LLTI are less likely to experience hot and extreme summer days.

**Table 4:** Associations between health or sociodemographic area indicator quintiles and projected numbers of hot and extreme summer days.

	Hot Summer Days						Extreme Summer Days		
	2.5 °C scenario			4.0 °C scenario			4.0 °C scenario		
	exp(beta)	LCrI	UCrI	exp(beta)	LCrI	UCrI	exp(beta)	LCrI	UCrI
Socioeconomic status									
(Intercept)	5.853	5.716	5.992	15.378	15.154	15.602	2.288	2.204	2.377
1 − lowest prevalence	ref			ref			ref		
2	0.999	0.970	1.030	0.999	0.980	1.018	0.997	0.953	1.045
3	0.988	0.958	1.020	0.994	0.974	1.014	0.970	0.926	1.019
4	0.979	0.948	1.014	0.988	0.968	1.009	0.961	0.912	1.013
5 − highest prevalence	0.975	0.940	1.012	0.986	0.963	1.010	0.948	0.897	1.004

COPD hospital admissions									
(Intercept)	5.752	5.618	5.889	15.157	14.941	15.382	2.231	2.155	2.313
1 − lowest prevalence	ref			ref			ref		
2	1.015	0.985	1.047	1.013	0.994	1.033	1.015	0.971	1.062
3	1.002	0.971	1.034	1.006	0.987	1.026	1.000	0.954	1.046
4	1.008	0.975	1.042	1.012	0.991	1.033	0.999	0.953	1.047
5 − highest prevalence	1.005	0.969	1.041	1.009	0.985	1.032	0.988	0.938	1.043

CHD hospital admissions									
(Intercept)	5.811	5.669	5.956	15.278	15.046	15.523	2.254	2.172	2.339
1 − lowest prevalence	ref			ref			ref		
2	0.994	0.965	1.024	0.998	0.979	1.018	0.992	0.948	1.039
3	0.994	0.964	1.027	1.000	0.980	1.020	0.982	0.937	1.029
4	0.998	0.964	1.034	1.003	0.981	1.026	0.982	0.930	1.035
5 − highest prevalence	0.993	0.956	1.032	1.000	0.976	1.025	0.983	0.927	1.042

<75 Preventable deaths									
(Intercept)	5.833	5.704	5.966	15.297	15.078	15.522	2.262	2.181	2.348
1 − lowest prevalence	ref			ref			ref		
2	0.989	0.960	1.019	0.995	0.977	1.014	0.984	0.940	1.029
3	0.996	0.966	1.026	1.003	0.983	1.023	0.987	0.942	1.033
4	0.991	0.959	1.022	0.999	0.979	1.020	0.982	0.936	1.032
5 − highest prevalence	0.986	0.953	1.021	0.996	0.973	1.019	0.969	0.915	1.023

Circulatory disease deaths									
(Intercept)	5.813	5.683	5.947	15.294	15.079	15.513	2.251	2.174	2.333
1 − lowest prevalence	ref			ref			ref		
2	0.997	0.968	1.028	0.999	0.981	1.018	0.999	0.955	1.045
3	0.993	0.963	1.024	0.998	0.979	1.018	0.990	0.945	1.036
4	0.992	0.962	1.023	0.997	0.977	1.018	0.984	0.937	1.031
5 − highest prevalence	0.996	0.962	1.030	1.000	0.979	1.022	0.983	0.933	1.035

CHD deaths									
(Intercept)	5.815	5.679	5.946	15.267	15.057	15.488	2.251	2.170	2.334
1 − lowest prevalence	ref			ref			ref		
2	0.998	0.970	1.028	1.003	0.985	1.022	1.002	0.956	1.048
3	0.995	0.964	1.024	1.000	0.981	1.019	0.987	0.942	1.035
4	0.993	0.962	1.025	1.000	0.980	1.021	0.985	0.936	1.035
5 − highest prevalence	0.991	0.956	1.028	1.000	0.977	1.023	0.981	0.928	1.037

Respiratory disease deaths									
(Intercept)	5.803	5.672	5.940	15.266	15.045	15.486	2.251	2.174	2.334
1 − lowest prevalence	ref			ref			ref		
2	1.001	0.972	1.031	1.001	0.982	1.020	0.997	0.954	1.043
3	0.998	0.969	1.027	1.003	0.984	1.022	0.991	0.947	1.039
4	0.999	0.967	1.029	1.004	0.984	1.025	0.991	0.943	1.043
5 − highest prevalence	0.988	0.953	1.022	0.996	0.975	1.018	0.972	0.922	1.026

Stroke deaths									
(Intercept)	5.793	5.669	5.918	15.279	15.073	15.491	2.243	2.163	2.322
1 − lowest prevalence	ref			ref			ref		
2	1.001	0.973	1.032	1.002	0.983	1.021	0.995	0.950	1.040
3	0.993	0.963	1.024	0.997	0.977	1.016	0.993	0.946	1.042
4	0.998	0.969	1.029	0.998	0.979	1.018	0.994	0.949	1.044
5 − highest prevalence	1.003	0.971	1.038	1.003	0.982	1.024	0.991	0.944	1.043

Limiting long term illness or disability (LLTI)									
(Intercept)	5.998	5.857	6.147	15.605	15.364	15.856	2.360	2.270	2.454
1 − lowest prevalence	ref			ref			ref		
2	0.999	0.972	1.028	0.999	0.981	1.017	0.997	0.956	1.040
3	0.977	0.945	1.008	0.985	0.965	1.006	0.965	0.918	1.015
4	0.958	0.924	0.993	0.974	0.951	0.997	0.937	0.885	0.991
5 − highest prevalence	0.894	0.854	0.933	0.937	0.911	0.963	0.842	0.788	0.900

Non-white population									
(Intercept)	5.480	5.315	5.653	14.699	14.425	14.979	2.086	1.984	2.184
1 − lowest prevalence	ref			ref			ref		
2	1.053	1.014	1.094	1.037	1.014	1.062	1.083	1.021	1.153
3	1.068	1.027	1.111	1.048	1.021	1.073	1.095	1.032	1.168
4	1.075	1.030	1.122	1.053	1.025	1.081	1.090	1.023	1.166
5 − highest prevalence	1.083	1.032	1.135	1.059	1.025	1.091	1.086	1.011	1.173

LCrI = Lower 95 % Credible interval, UCrI = Upper 95 % Credible interval. Note: The paucity of projected number of extreme hot days under the 2.5 °C scenario prevented the modelling of this scenario, due to convergence, and poor model diagnostics.

## 5 Discussion

### 5.1 Key findings

Health inequalities exist whereby health outcomes are poorer in deprived, non-white and older populations, and those with pre-existing health conditions. Within England there is also a north–south divide whereby health outcomes are worse in the North, driven by an array of social, demographic, political and environmental factors (Bambra et al., 2014). This study linked national climate data to small geographical areas to investigate the extent to which projected climate change will vary geographically and by population sub-group, and whether these variations are likely to increase or reduce existing health inequalities. There were clear geographical variations, whereby the South of England currently experiences the highest number of hot and extreme summer days and will experience the largest increases in both. These geographical variations drive some of the observed demographic and socioeconomic variations. Compared with the North of England, the South is characterised by lower deprivation, lower prevalence of chronic diseases, and higher prevalence of ethnic minority groups; therefore, these groups will experience larger increases in hot and extreme summer days. Finally, the increase in the number of hot and extreme days was predicted to be lower in older age-groups. Together, the findings suggest that, apart from ethnicity, the predicted changes in extreme heat exposure will impact sub-groups of the population who currently have better health outcomes. However, all populations will experience significance increases in both hot and extreme summer days based on global warming scenarios.

### 5.2 Comparison with other literature

Previous research has shown regional differences in vulnerability to heat-related mortality, with London and the East Midlands identified as the most susceptible regions (Hajat et al., 2014). This underscores two critical points: first, London, which currently has and is also projected to have the highest number of hot and extreme summer days, is also the most vulnerable to heat-related mortality; second, regions not experiencing the highest number of hot and extreme summer days might still exhibit significant vulnerability due to lack of heat adaptation. Heat adaption may be implemented through various means, including behavioural changes, environmental interventions, and building interventions, however there is a lack of evidence relating to whether this adaptation is equal across population subgroups (Arbuthnott et al., 2016). Further, small-area analysis of temperature-mortality relationships suggests that the Minimum Mortality Temperature (effectively the optimum temperature, above which mortality rises) is typically lower in the north and west of England, and highly variable even within cities (Gasparrini et al., 2022). These findings emphasise the need for climate adaptation strategies that consider regional and demographic contexts within the country to address social and health outcomes effectively.

Globally, studies have examined the varying effects of climate change on population health, identifying specific global regions more susceptible to its negative impacts (Pfahl et al., 2017, Samson et al., 2011). Additionally, global models have assessed the total populations at risk under different global heating scenarios (Gasparrini et al., 2017). However, there is limited evidence within countries that highlights regional disparities across sociodemographic characteristics, including climate-sensitive health outcomes and climate-health projections (Harper et al., 2021). In the UK, most research on the future impact of heat has concentrated on mortality outcomes (Hajat et al., 2014, Murage et al., 2024b), often overlooking additional factors such as risk of hospitalisation and health inequalities. Our results indicate that the most socioeconomically deprived areas will not experience the highest number of hot and extreme summer days across various global heating projections, compared to less deprived areas, although they will experience a similar increase in hot and extreme summer days from baseline due to global heating. However, heat-exposure is just one of the three key vulnerability pathways contributing to longstanding structural inequalities that impact on health inequity (Smith et al., 2022). It is therefore crucial to consider other vulnerabilities that may increase health inequalities, such as sensitivity and resilience to the increasing projected number of hot and extreme summer days, which would ultimately increase vulnerability due to factors such as lack of adaptation to climate change (housing quality, air conditioning, occupation), pre-existing heat-sensitive medical conditions and multi-morbidity, that contribute to health inequalities. Therefore, even though the most deprived areas may have a lower projected total number of hot and extreme summer days, their greater vulnerability and reduced capacity to adapt suggest they will likely face worse health outcomes.

Although our findings indicate that areas with greater health and social needs experience fewer hot and extreme summer days, further evidence from England suggests that hospital admissions increase disproportionately among older adults and socioeconomically deprived populations during extreme heat. In particular, admissions for injuries, respiratory, infectious, and metabolic diseases rise significantly when temperatures exceed 30 °C (Rizmie et al., 2022). Similarly, in Brazil, socioeconomic inequalities are evident in heat-related hospitalisations for conditions such as ischemic heart disease, asthma, pneumonia, renal diseases, mental health disorders, and neoplasms (Xu et al., 2020). These findings highlight a critical disparity: while global heating is more pronounced in southern England—an area that, at a population inequality level, tends to have comparably better health outcomes than its northern counterparts (Munford et al., 2023) —regions where temperature increases are geographically uniform may experience a widening of socioeconomic and health inequalities due to disproportionate heat-related health impacts on vulnerable populations.

Similar to studies in North America (Gronlund, 2014, Hsu et al., 2021), we found that areas with a greater proportion of minority ethnic populations will experience a greater number of hot and extreme summer days. This finding is likely strongly related to London being the region experiencing the greatest heat impact in England, as well as being projected to experience the largest temperature increases, and also having the most ethnically diverse population (and lowest proportion from a White background) across all English regions (London: 21 % Asian, 14 % Black, 6 % mixed, 54 % white) (UK Government, 2022). It is important to note that minority ethnic populations may also experience greater disadvantage to hot and extreme summer days through other social and economic factors that may reduce their capacity to adapt, such as insecure work, lower income, poor housing quality and pre-existing physical and mental illness (Nazroo, 2022).

Although we did not include mental health conditions, systematic reviews demonstrate an increased risk of suicide associated with high ambient temperatures, as well as evidence for increase in hospital admissions due to mental illness during heatwave periods (Thompson et al., 2023) and heat-related morbidity and mortality among people with known mental health problems (Thompson et al., 2018). Given the significant overlap between physical and mental health conditions, further research is needed to quantify the predicted mental health impacts so that health and social services can be better prepared and prevent further widening of health inequalities.

### 5.3 Strengths and limitations

Our study has a number of strengths. We utilised small-area climate projections based on global heating scenarios provided by the UK’s meteorological office. These data were mapped to small administrative units and integrated with health data from the UK Government's Office for Health Improvement & Disparities, leveraging robust mortality, secondary care, and census data. This comprehensive approach enabled us to conduct an equity analysis across nearly 7,000 small areas in England. Small area population statistics were joined to the climate data to provide accurate population level impacts by age group.

However, there are limitations to consider. Our health outcome data were at the area level, preventing us from examining the impact of climate change projections with individual-level adjustments, even though the hospitalisation and mortality data were age and sex standardised. A limitation of this study is the reliance on absolute temperature thresholds (30 °C for Hot Summer Days and 35 °C for Extreme Summer Days) calculated by the UK Met Office, which, while based on robust historical and projected data for small geographical areas, may not fully capture localised variability or other thresholds relevant to specific populations or regions and subsequent health outcomes. Additionally, since climate projections are not tied to specific future dates (i.e., we do not know precisely when global heating might reach 2.5 °C or 4.0 °C), we could not accurately link them to future population projections. Therefore, our analysis relied on the current small-area geographies of population age distribution, socio-economic deprivation, ethnicity and health for England.

## 6 Conclusions

Regional differences within England are evident in the projected number of hot and extreme summer days, with a 4.0 °C increase in global heating significantly increasing the number of people experiencing hot and extreme summer days annually from the 2001 to 2020 baseline. A 2.5 °C increase will mean that 16 million people would experience 10 or more hot summer days per years, which would increase to 43 million for a 4.0 °C increase, at baseline no area experiences 10 or more hot summer days. The increase in hot and extreme summer days will disproportionately affect areas with greater ethnic populations, however this relationship is in the opposite direction for areas that currently experience better health outcomes. Therefore, global heating is likely to worsen overall health and widen ethnic inequalities in health, but may reduce or increase other types of health inequalities depending on the complexity of temperature change geographies and interaction with multiple vulnerability pathways.

Our findings underscore the urgent need for climate mitigation and adaption strategies on two levels: (1) local strategies to support populations, especially vulnerable sociodemographic groups, in coping with the increased number of hot and extreme summer days due to global heating, and (2) governmental and global strategies to prevent global temperatures from exceeding 2.5 °C or reaching 4.0 °C, given the severe population-level health impacts such increases would entail.

## 7 Funding information

JO & NN are employed by the MRC/CSO Social and Public Health Sciences Unit, University of Glasgow, and supported by the Medical Research Council [grant number MC_UU_00022/4] and Chief Scientist Office [grant number SPHSU19]. The researchers were independent of the funders; the funders had no role in the study design, data collection, analysis and interpretation of data, the decision to publish, or the preparation of the manuscript.

## 8 Rights retention statement:

For the purpose of open access, the author(s) has applied a Creative Commons Attribution (CC BY) licence to any Author Accepted Manuscript version arising from this submission.

## CRediT authorship contribution statement

**Jonathan R Olsen:** Writing – original draft, Visualization, Methodology, Formal analysis, Conceptualization. **Claire Niedzwiedz:** Writing – review & editing, Investigation. **Natalie Nicholls:** Writing – review & editing, Formal analysis. **Benedict W Wheeler:** Writing – review & editing, Investigation. **Frederick K Ho:** Writing – review & editing, Investigation. **Jill P. Pell:** Writing – review & editing, Investigation, Conceptualization.

## Declaration of competing interest

The authors declare that they have no known competing financial interests or personal relationships that could have appeared to influence the work reported in this paper.

## Data Availability

The authors do not have permission to share data.
