# Supplementary material for: Sociodemographic and geographic inequalities in exposure to projected hot and extreme summer days in England: A nationwide socio-spatial analysis
Source: Environ Int. 2025 Mar;197:109351. doi: 10.1016/j.envint.2025.109351 (PMC12316637; doi:10.1016/j.envint.2025.109351)
Supplement: Supplementary Data 1 [file mmc1.docx]

**Sociodemographic and geographic inequalities in exposure to projected hot and extreme summer days in England: a nationwide socio-spatial analysis.**

**Supplementary Materials**

***Supplementary Table 1: English population experiencing hot and extreme summer days (annual number) in millions at baseline and for a 2.5°C and 4.0°C global heating temperature increase, by age group.***

| **Annual number of days** | **Under 5**  **(Number (column %))** | **5 to 64**  **(Number (column %))** | **65 and over**  **(Number (column %))** |  |
| --- | --- | --- | --- | --- |
|  |  |  |  |  |
| **(a) Hot summer days (days >30°C): 2.5°C** | | | |  |
| 9 or less | 1,993,582 (68.6) | 28,438,912 (69.2) | 7,829,379 (76.3) |  |
| 10 or more | 912,799 (31.4) | 12,633,127 (30.8) | 2,431,064 (23.7) |  |
| *Chi square* | p<0.001 | | |  |
| **(b) Hot summer days (days >30°C): 4.0°C** | | | |  |
| 9 or less | 586,760 (20.2) | 8,473,630 (20.6) | 2,422,351 (23.6) |  |
| 10 or more | 2,319,621 (79.8) | 32,598,409 (79.4) | 7,838,092 (76.4) |  |
| *Chi square* | p<0.001 | | |  |
| **(c) Extreme summer days (days >35°C): 2.5°C** | | | |  |
| 1 or less | 2,726,385 (93.8) | 38,560,663 (93.9) | 9,706,907 (94.6) |  |
| 2 or more | 179,996 (6.2) | 2,511,376 (6.1) | 553,536 (5.4) |  |
| *Chi square* | p<0.001 | | |  |
| **(d) Extreme summer days (days >35°C): 4.0°C** | | | |  |
| 1 or less | 439,917 (15.1) | 6,518,013 (15.9) | 1,996,278 (19.5) |  |
| 2 or more | 2,466,464 (84.9) | 34,554,026 (84.1) | 8,264,165 (80.5) |  |
| *Chi square* | p<0.001 | | |  |

***Supplementary Table 2: Baseline and projected number of hot summer days by area-level rates of hospital admissions, mortality ratios and sociodemographic factors for a 2.5c and 4.0c global heating increase: lowest & highest condition specific quintiles reported.***

| **Number of hot summer days** | **Area-level condition prevalence** | | | | | |
| --- | --- | --- | --- | --- | --- | --- |
|  | **Least** | | | **Most** | | |
|  | **Baseline** | **2.5c increase (% increase from baseline)** | **4.0c increase (% increase from baseline)** | **Baseline** | **2.5c increase (% increase from baseline)** | **4.0c increase (% increase from baseline)** |
| COPD hospital admissions | 3 | 8 (148) | 19 (500) | 2 | 6 (136) | 14 (502) |
| CHD hospital admissions | 4 | 9 (148) | 21 (483) | 2 | 5 (133) | 14 (496) |
| <75 Preventable deaths | 3 | 8 (149) | 19 (493) | 2 | 5 (136) | 13 (515) |
| Circulatory disease deaths | 3 | 9 (147) | 20 (481) | 2 | 6 (137) | 14 (508) |
| CHD deaths | 3 | 9 (148) | 20 (483) | 2 | 5 (135) | 14 (506) |
| Respiratory disease deaths | 3 | 8 (146) | 20 (486) | 2 | 5 (136) | 14 (509) |
| Stroke deaths | 4 | 9 (143) | 20 (472) | 2 | 6 (140) | 15 (506) |
| Socioeconomic status | 3 | 8 (148) | 19 (489) | 2 | 5 (134) | 14 (503) |
| LLID | 4 | 10 (142) | 23 (449) | 2 | 4 (140) | 11 (577) |
| Non-white population | 2 | 4 (151) | 12 (588) | 4 | 10 (132) | 23 (427) |

***Supplementary Table 3: Baseline and projected number of hot summer days by area-level rates of hospital admissions, mortality ratios and sociodemographic factors for a 2.5c and 4.0c global heating increase: lowest & highest condition specific quintiles reported.***

| **Number of extreme summer days** | **Area-level condition prevalence** | | | | | |
| --- | --- | --- | --- | --- | --- | --- |
|  | **Least** | | | **Most** | | |
|  | **Baseline** | **2.5c increase (% increase from baseline)** | **4.0c increase (% increase from baseline)** | **Baseline** | **2.5c increase (% increase from baseline)** | **4.0c increase (% increase from baseline)** |
| COPD hospital admissions | 0.2 | 0.5 (196) | 3.1 (1699) | 0.1 | 0.3 (147) | 2.2 (1700) |
| CHD hospital admissions | 0.2 | 0.6 (189) | 3.6 (1595) | 0.1 | 0.3 (151) | 2.1 (1716) |
| <75 Preventable deaths | 0.2 | 0.5 (192) | 3.3 (1649) | 0.1 | 0.3 (150) | 2 (1782) |
| Circulatory disease deaths | 0.2 | 0.6 (189) | 3.4 (1617) | 0.1 | 0.3 (150) | 2.2 (1727) |
| CHD deaths | 0.2 | 0.6 (193) | 3.4 (1643) | 0.1 | 0.3 (147) | 2.1 (1757) |
| Respiratory disease deaths | 0.2 | 0.5 (182) | 3.2 (1606) | 0.1 | 0.3 (155) | 2.1 (1791) |
| Stroke deaths | 0.2 | 0.5 (170) | 3.4 (1570) | 0.1 | 0.3 (164) | 2.3 (1722) |
| Socioeconomic status | 0.2 | 0.6 (199) | 3.3 (1668) | 0.1 | 0.3 (138) | 2.1 (1710) |
| LLID | 0.3 | 0.7 (182) | 4.0 (1487) | 0.1 | 0.2 (168) | 1.6 (2044) |
| Non-white population | 0.1 | 0.2 (175) | 1.8 (1971) | 0.3 | 0.6 (138) | 3.7 (1393) |

***Supplementary Figure 1: Index of Multiple Deprivation (IMD) Quintiles, England, 2019.***

***
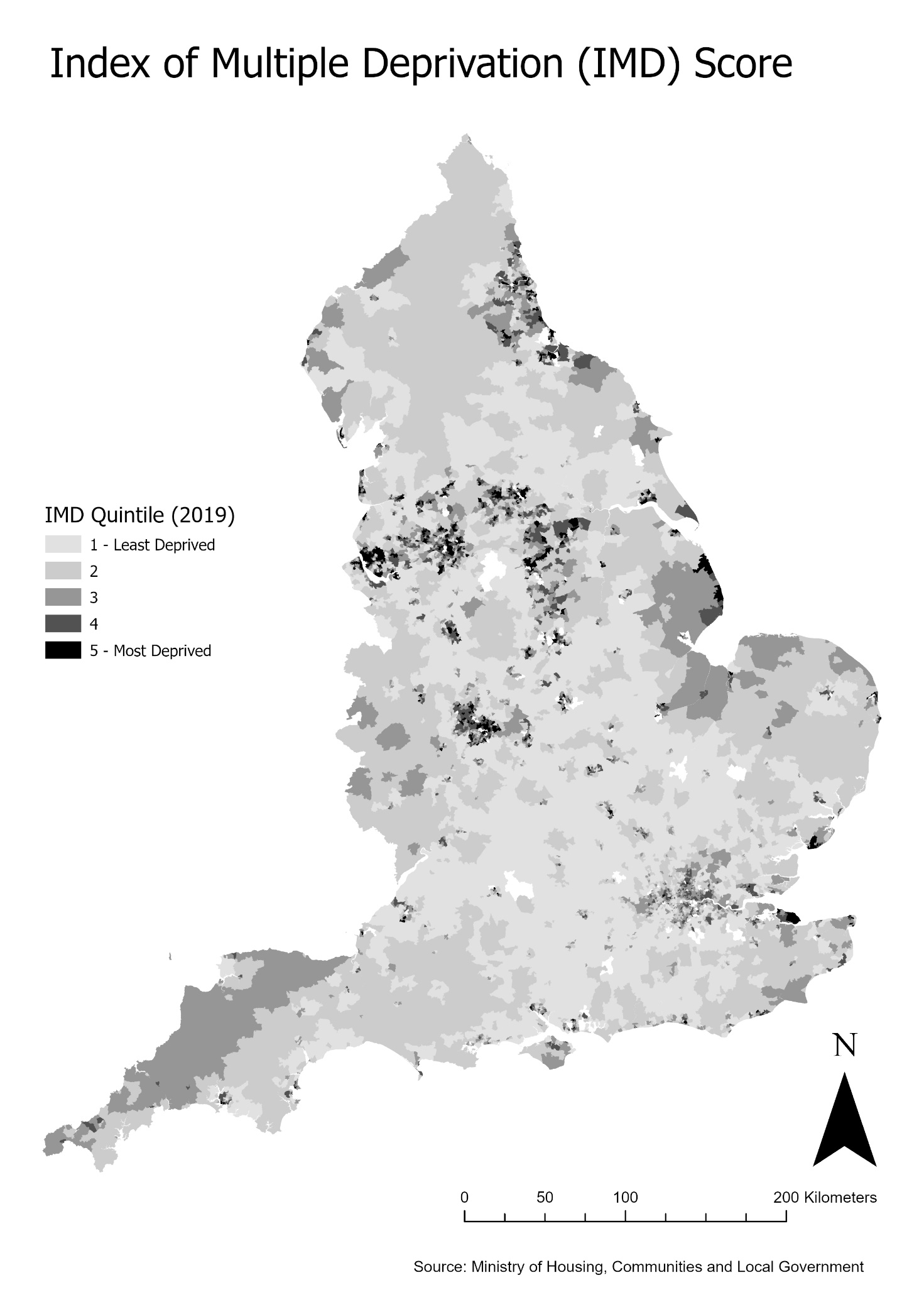
***

***Supplementary Figure 2: Baseline and projected number of extreme summer days by area-level rates of hospital admissions, mortality ratios and sociodemographic factors for a 2.5c and 4.0c global heating increase: lowest & highest condition specific quintiles reported.***

**
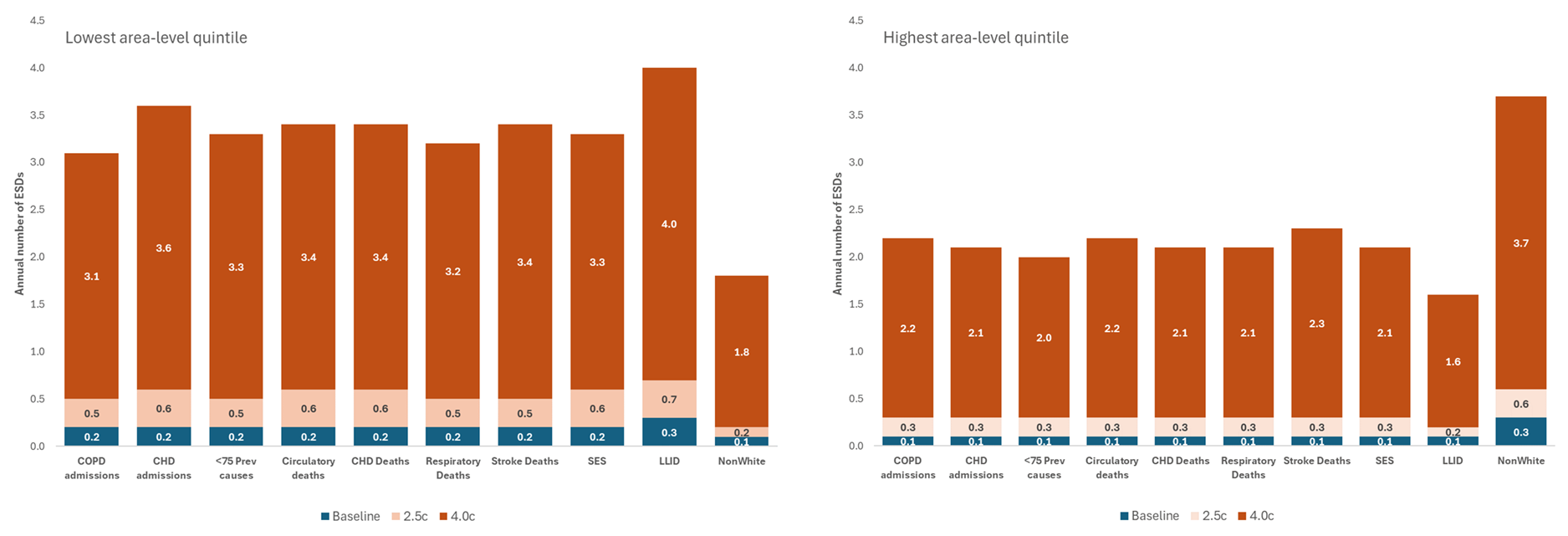
**

***Supplementary Figure 3: Mean number of hot and extreme summer days (annual number) of a 4.0°C global heating temperature increase by area-level emergency hospital admissions, mortality, and sociodemographic characteristics.***

***
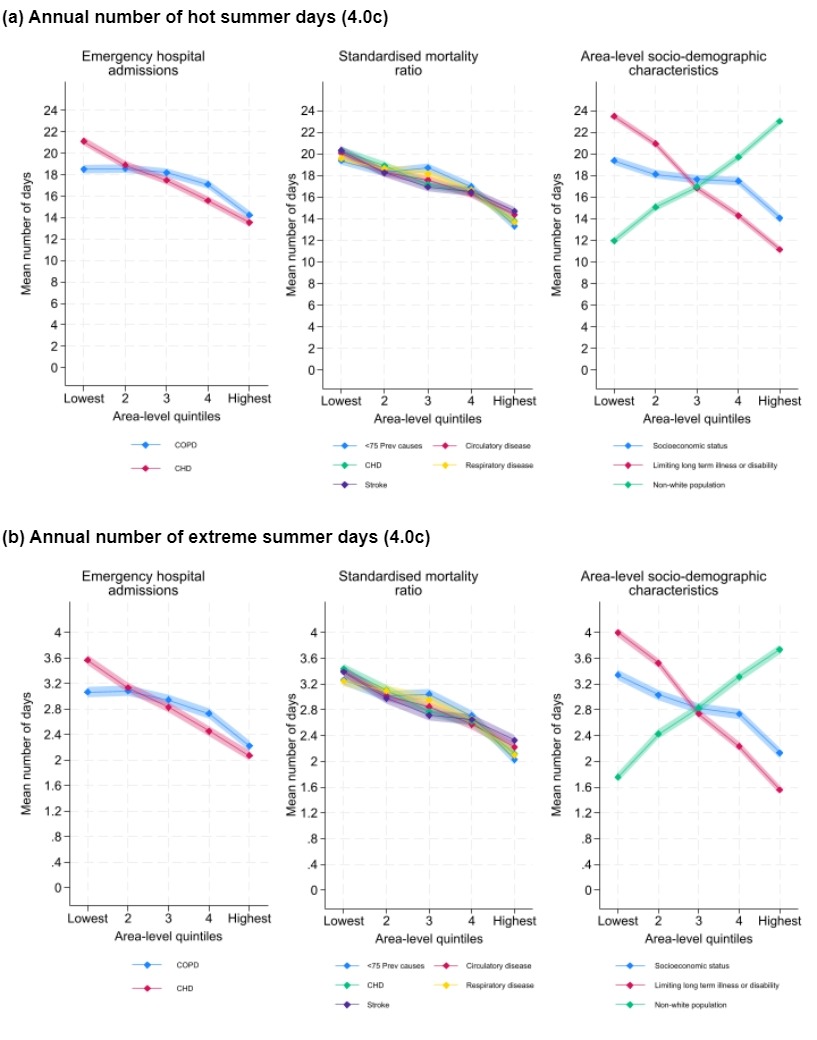
***
